# An Attention-Enhanced RegNetY Framework for Detection and Classification of Vertical Misfit in Implant-Supported Restorations: A Retrospective Study

**DOI:** 10.3390/diagnostics16111613

**Published:** 2026-05-25

**Authors:** Tuba Talo Yildirim, Aybike Cengiz Dagtekin, Nurullah Düger, Ayşe Rençber Kizilkaya, Furkan Talo, Emre Arslan, Mucahit Karaduman, Muhammed Yildirim

**Affiliations:** 1 Department of Periodontology, Faculty of Dentistry, Firat University, Elazig 23119, Turkey; nduger@firat.edu.tr (N.D.); e_arslan@firat.edu.tr (E.A.); 2 Department of Prosthodontics, Faculty of Dentistry, Firat University, Elazig 23119, Turkey; a.cengiz@firat.edu.tr (A.C.D.); arencber@firat.edu.tr (A.R.K.); 3 Digital Transformation and Software Office, Rectorate, Firat University, Elazig 23119, Turkey; ftalo@firat.edu.tr; 4 Department of Software Engineering, Malatya Turgut Ozal University, Malatya 44200, Turkey; mucahit.karaduman@ozal.edu.tr; 5 Department of Artificial Intelligence and Data Engineering, Firat University, Elazig 23119, Turkey; muhammedyildirim@firat.edu.tr

**Keywords:** artificial intelligence, dental implant, implant-supported dental prosthesis, neural network, vision system

## Abstract

**Background/Objectives**: The aim of this study is to test different convolutional neural network (CNN) and Transformer-based models to detect and classify vertical misfit at the abutment-prosthesis interface on panoramic radiographs, and to develop a hybrid deep learning model enhanced with attention mechanisms. **Methods**: A dataset consisting of a total of 566 images, manually classified as 249 ‘fit’ and 317 ‘misfit’ cases by two experts, was created. Images were resized to 224 × 224 and divided into training, validation, and test groups. The deep learning model yielding the most successful results was determined as the backbone; a hybrid model was developed by integrating three different attention modules (SE, CBAM, and ECA) into this structure. Model performance was evaluated using accuracy, precision, sensitivity, and F1 score metrics. **Results**: CNN-based models (RegNetY-800MF, ConvNeXt-Tiny, EfficientNetV2-S, ResNet50) performed better than Transformer-based models (DeiT, Swin-Tiny) in all metrics. The proposed hybrid model exhibited the highest success among all tested models with a 99.12% accuracy rate. This model reached a 100% precision value in the misfit group and yielded no false positive results. The F1 scores of the hybrid model were recorded as 99.01% for the fit group and 99.21% for the misfit group. **Conclusions**: The findings of this study demonstrate that attention-enhancing deep learning frameworks have the potential to significantly improve the diagnostic utility of routine panoramic radiographs. It shows that panoramic imaging, when supported by advanced artificial intelligence, can provide valuable diagnostic support in detecting vertical misfit. The developed model has the potential to become a reliable clinical decision support system.

## 1. Introduction

Today, dental implants have become one of the most common treatment procedures for replacing missing teeth due to high survival rates and predictable clinical outcomes [1,2]. The long-term success of these treatments depends not only on ensuring osseointegration but also on the biomechanical stability of the prosthetic restoration and the passive fit between implant components [3,4]. However, achieving absolute fit is not always possible due to fabrication errors and the nature of clinical processes [5].

Gaps ranging from 10 to 150 μm on the vertical axis in components between the dental implant and the prosthetic superstructure are evaluated within clinically tolerable limits in the literature [4,6,7]. Deviations exceeding this threshold are defined as vertical misfit and are accepted as a risk factor threatening the prognosis of implant treatment [4]. Micro-gaps resulting from the incompatibility between the implant and prosthesis act as a reservoir for plaque accumulation, leading to bacterial leakage [7,8]. Mechanically, this condition can lead to stress concentration in the screw and implant neck region, screw loosening, and material fatigue [3,8]. Biologically, it triggers marginal bone loss, causing torque loss and, in addition to mechanical effects, leads to complications such as implant or prosthesis loss in the worst-case scenario [7,9].

Although numerous methods have been described in the literature to evaluate the fit of prosthetic restorations, a standard protocol is not yet available [10]. At the intraoral examination stage, marginal integrity can be detected through visual inspection, probing, and radiographic analyses, as well as specific diagnostic approaches such as the Sheffield test (single screw test) or resistance test in screw-retained restorations [5,9,11]. These clinical methods vary depending on the clinician’s experience and are generally subjective [9]. Due to these limitations, radiographic evaluation is an indispensable tool [11,12].

Although periapical radiographs are frequently preferred in imaging the fit of the implant and prosthesis because they offer great detail, panoramic radiographs are more commonly used in clinical practice during general screening, treatment planning, and follow-up processes [12,13]. While panoramic radiographs have the advantage of visualizing the entire dental arch, adjacent anatomical structures, and implants in a single image, they possess the disadvantages of lower resolution, distortion, and superposition compared to periapical images [11,12]. In particular, these structural distortions in panoramic images make the detection of micron-level marginal misfits by the human eye difficult and reduce diagnostic reliability. Conversely, variations are observed in periapical radiographs depending on the shooting angle [14].

In recent years, artificial intelligence algorithms have shown high success in many areas of implantology [15,16,17]. The limited ability of the human eye to distinguish these fine micron-level details in radiography has created a need for AI-supported automatic diagnostic systems [7,18,19]. Nevertheless, studies on the detection of vertical misfit at the implant-prosthesis interface are quite limited in the literature. Recently, Fasih et al. reported a 92.7% accuracy rate in detecting vertical misfit at the abutment-prosthesis interface using the ResNet-50 architecture [19]. Although being a pioneer in this field, they conducted this study using a single convolutional neural network (CNN) model for detection on periapical images. Standard CNN models tend to focus on global features in the image; therefore, they may remain insufficient in capturing micron-level pixel details like those at the abutment-prosthesis interface. Attention mechanisms, which are among current approaches developed to capture details, enable the model to suppress unnecessary background noise and focus on critical regions [20].

The aim of this study is to test different CNN and Transformer-based models to detect and classify vertical misfit at the abutment-prosthesis interface on a new dataset created on panoramic radiographs, and to develop a hybrid deep learning model enhanced with Attention mechanisms. Our hypothesis is that the proposed hybrid model can detect misfit cases on panoramic radiographs with much higher sensitivity compared to standard models.

## 2. Materials and Method

### 2.1. Data Collection and Ethical Approval

This retrospective study was approved by the Firat University Non-Interventional Ethics Committee (FUGOEK) (No. 2025-17-07) and was conducted in accordance with the Declaration of Helsinki. A total of 500 panoramic images obtained from patients who applied to the Fırat University Faculty of Dentistry between January 2022 and September 2025 were examined. Our study was conducted on a patient-by-patient basis. Furthermore, 566 images from 500 patients were used in the study. All images were obtained from different implant regions. All scans were acquired using a CS 8100 unit (Carestream Dental LLC, Atlanta, GA, USA) under standard exposure protocols (68 kV, 8 mA, 66 mGy·cm^2^, 10.8 s). The images were saved in PNG format and processed using ImageJ software (version 1.54p, National Institutes of Health, Bethesda, MD, USA).

All radiographic data utilized in this study were obtained retrospectively from patients who had previously provided written informed consent for the anonymous use of their data for scientific purposes. This consent was obtained during the initial admission to the clinic; therefore, no additional informed consent was required. The data were anonymized to prevent the identification of patients.

### 2.2. Inclusion-Exclusion Criteria and Dataset

Inclusion criteria comprised panoramic radiographic images of patients who had undergone final prosthetic rehabilitation on dental implants. No restrictions were imposed regarding implant brand, crown material type, patient age, gender, or clinician. Exclusion criteria included images of low quality due to exposure errors, artifacts, superposition, or distortion.

**Figure 1:**
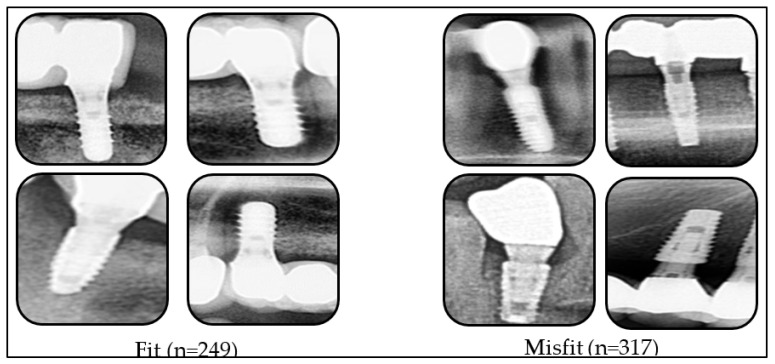
Sample images and numbers for each class.

A dataset consisting of 566 images, manually classified as ‘fit’ and ‘misfit’ by two experts (A.C.D, N.D), was established. To prevent data leakage, the dataset was divided by patient, not by image. First, all panoramic image segments of the same patient were numbered and grouped. Then, they were separated into training, validation, and test datasets. This approach ensured that images from the same patient did not appear in different datasets, allowing for a more reliable evaluation of model performance. The created dataset and the distribution of samples are presented in Figure 1.

### 2.3. Manual Grouping and Standardization

The classification of the abutment-prosthesis relationship as “fit” or “misfit” was performed independently by two experts (N.D., A.C.D.) in implant surgery and prosthodontics. To determine inter-observer and intra-observer consistency, 100 panoramic radiographs with implant-supported fixed restorations (Fit [*n* = 50], Misfit [*n* = 50]) were selected, and manual assessments were repeated two weeks later. Images on which the two experts could not reach a consensus were excluded from the study.

The reliability of the measurements was assessed using intraclass correlation coefficients (ICCs) to evaluate inter-observer and intra-observer consistency. ICC values of 0.9841 and 0.9878 were observed for height and width measurements, respectively, indicating excellent intra-observer reliability. Furthermore, ICC values of 0.9745 and 0.9766 were also observed, demonstrating excellent inter-observer reliability. These results confirm the accuracy and consistency of the measurement process, as evidenced by the strong reliability of measurements taken by different operators and over time.

### 2.4. Training and Proposed Model

The images were initially resized to 224 × 224 pixels to meet the input requirements of all models. Experiments were conducted in the Google Colab environment utilizing an Tesla T4 GPU (NVIDIA, Santa Clara, CA, USA). The dataset was partitioned into Training, Validation, and Test sets with a ratio of 70/10/20, respectively. Data augmentation was not performed in this study. The study was conducted using original data. Accuracy, precision, recall, and F1-score metrics were employed to evaluate model performance. Experiments were carried out by fine-tuning all models for 10 epochs, and performance comparisons were conducted. During the training process, the learning rate was set to 1 × 10^−4^, the batch size to 16, and the AdamW optimization algorithm was utilized with the Cross-Entropy loss function. Final performance results were reported based on the test data.

**Figure 2:**
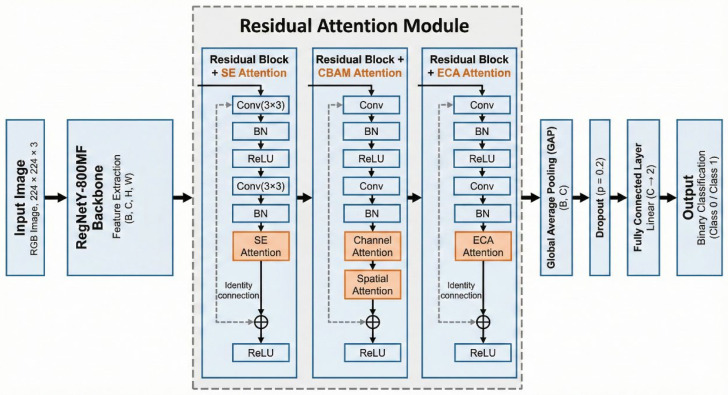
Flowchart of the proposed method.

The general flow diagram of the proposed model for the classification of the original fit/misfit dataset is presented in Figure 2. The general architecture of the proposed model consists of a backbone and attention mechanisms. The backbone yielding the most successful results was selected to be used as a feature extractor, followed by classification through feature enhancement using attention mechanisms.

RegNetY-800MF, ConvNeXt-Tiny, EfficientNetV2-S, ResNet50, DeiT, and Swin-Tiny models were utilized for backbone selection for the proposed method. The RegNetY-800MF model, which provided the most successful results, was determined as the backbone. The RegNetY-800MF model was selected as the backbone model after evaluating multiple performance metrics. Features extracted from this model serve as input to the module designated as the Residual Attention Block. This module incorporates the Squeeze-and-Excitation (SE) Attention Module, Convolutional Block Attention Module (CBAM), and Efficient Channel Attention Module (ECA) [21,22,23]. The proposed model utilizes multiple attentional mechanisms together, enabling it to better capture complementary feature representations by modeling channel-based dependencies (SE, ECA) and spatial attention (CBAM) simultaneously. Each attention module includes convolution, batch normalization, and ReLU mechanisms. These processes stabilize the model’s training process and also increase the nonlinear representational power of the proposed model. Features passing through these stages undergo attention mechanisms and are combined with the features arriving via the residual connection. Thus, important features are enhanced while information loss is prevented. Residual attentions play a critical role in preserving low-level structural information and reducing gradient loss, facilitating deeper feature refinement.

Features passing through three different attention blocks are subjected to Global Averaging Pooling (GAP) and Dropout. GAP reduces spatial dimensions while preserving distinct global identifiers. Dropout (*p* = 0.2) is used to prevent overfitting due to the small size of the dataset. Finally, class prediction is performed using the fully connected layer. The fully connected layer performs the final mapping of high-level abstract features to class probabilities, enabling effective differentiation between suitable and unsuitable cases. Furthermore, the proposed model uses both local structural details and global contextual information to achieve successful classification performance.

## 3. Results

A hybrid deep learning model enhanced with attentional mechanisms has been developed to detect and classify vertical discrepancies at the abutment-prosthesis interface in panoramic radiographs. The developed model was compared with different CNN and ViT models accepted in the literature. The performance of the models was evaluated using different performance measurement metrics. The application results were obtained in the Google Colab environment using an NVIDIA Tesla T4 GPU. The performance metrics of all models used in our study are presented in Table 1.

In this study, 4 CNN-based and 2 Transformer-based models were utilized. CNN-based models demonstrated superior performance across all metrics compared to Transformer-based models (DeiT, Swin-Tiny). The models exhibiting the highest accuracy rates, in descending order, were RegNetY-800MF, ConvNeXt-Tiny, EfficientNetV2-S, ResNet50, DeiT, and Swin-Tiny.

The best-performing model, RegNetY-800MF (98.25%), was enhanced with Attention mechanisms to form the proposed hybrid model. The proposed model outperformed all other models with an accuracy rate of 99.12%. The classification-based performance of the proposed model is provided in Table 2.

The proposed model achieved an F1 score of 99.01% for the fit group and 99.21% for the misfit group. The model made no errors in the misfit group, achieving a precision of 100% (False Positive = 0).

**Figure 3:**
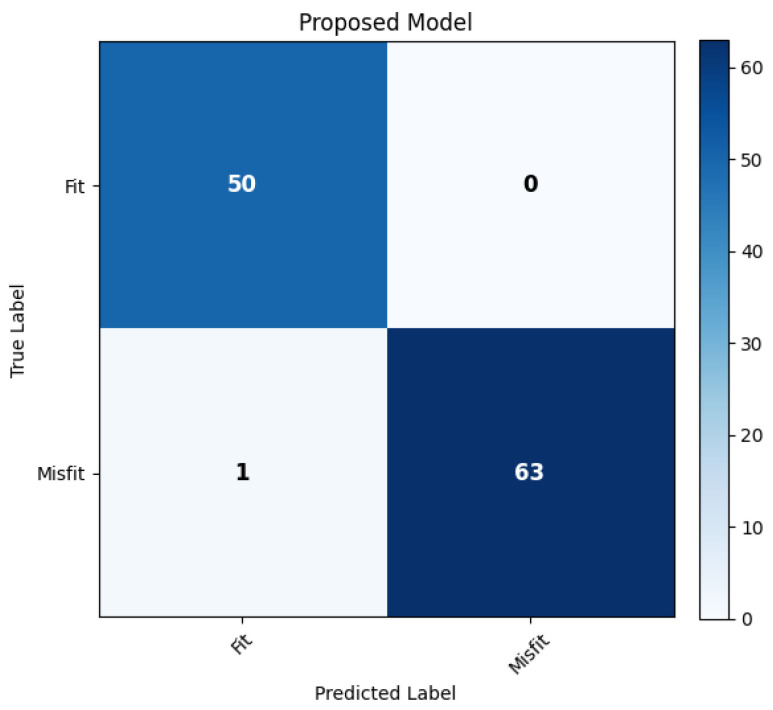
Confusion matrix for the proposed model.

The confusion matrix for the proposed model is shown in Figure 3.

While the proposed model misclassified only 1 case in the fit group, it made no misclassifications in the misfit group. The results obtained when the fit-misfit classification performance of the proposed model was evaluated with 95% confidence intervals are presented in Table 3.

**Figure 4:**
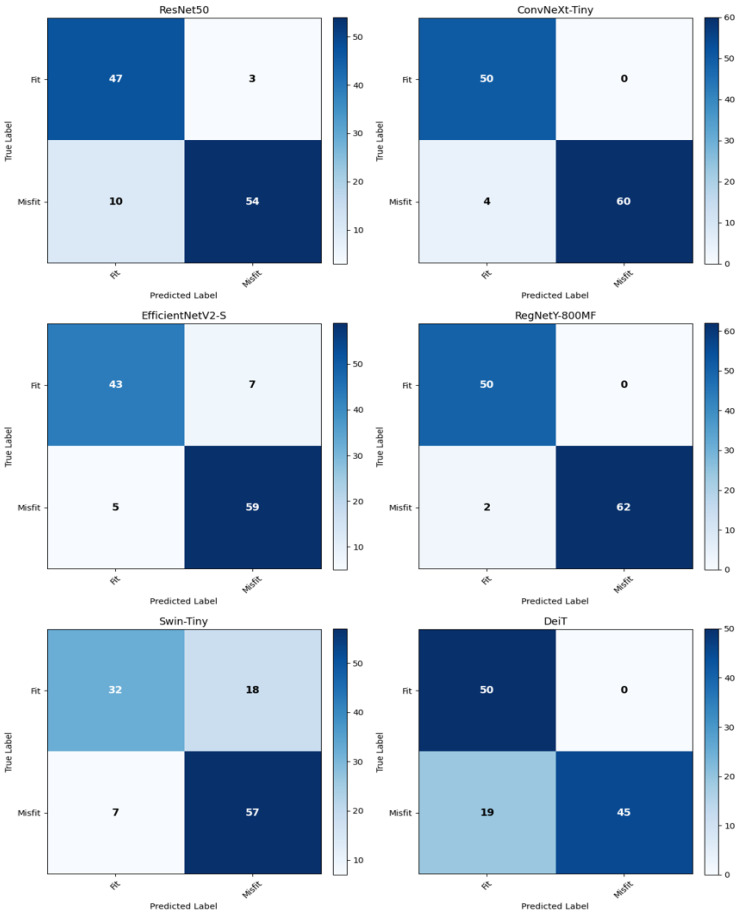
Confusion matrices for all architectures.

The fact that the proposed model achieved a 100% recall rate demonstrates its ability to detect clinically critical discrepancies without missing any. The narrow range of confidence intervals indicates that the proposed model’s performance is stable across different samples. The results show that the proposed model offers a reliable structure for clinical decision support systems, in addition to its high discrimination power. Confusion matrices for all models are presented in Figure 4.

When Figure 4 is examined, the ResNet50 model correctly predicted 101 out of 114 test images, while it incorrectly predicted 13 test images. The ConvNext-Tiny model incorrectly predicted 4 out of 114 test images. RegNetY-800MF incorrectly predicted only 2 test images. The EfficientNetV2-S model incorrectly predicted 12 test images, while Swin-Tiny incorrectly predicted 25 test images and DeiT incorrectly predicted 19 test images. Among the pre-trained models, the RegNetY-800MF model was the most successful. The least successful model among those used in the study was DeiT. When the number of correct and incorrect predictions is examined, CNN-based models are seen to provide a more balanced classification. In particular, RegNetY-800MF and ConvNeXt-Tiny achieved successful results by exhibiting low false positive and false negative rates. Transformer-based models, on the other hand, reached a higher number of false classifications. This indicates that these architectures may have difficulty effectively capturing local features in panoramic radiographs.

## 4. Discussion

Vertical misfit at the prosthesis-abutment interface leads to complications such as peri-implant mucositis, peri-implantitis, crown loss, and implant loss [4,7]. The aim of this study was to develop a high-precision artificial intelligence model for the detection of vertical misfit at the abutment-prosthesis interface using challenging imaging modalities containing distortion and low resolution, such as panoramic radiographs. The findings indicated that the RegNetY-800MF model reinforced with attention mechanisms (SE, CBAM, ECA) performed better than both traditional CNN and Transformer architectures, achieving an accuracy rate of 99.12%.

There are limited studies on the detection of abutment-prosthesis misfit using deep learning. The only current study on this subject, conducted by Fasih et al., achieved 92.7% accuracy using the ResNet-50 model on periapical radiographs [19]. In our study, the standard ResNet-50 model achieved an accuracy of only 88.60% when tested on panoramic images. Considering that periapical radiographs offer greater detail compared to panoramic images, the performance drop of ResNet-50 on our dataset is an expected outcome [12]. However, the Attention-enhanced RegNetY model we developed represents a significant technical innovation by elevating the 92.7% success rate achieved by Fasih et al. with high-resolution periapical radiographs to 99.12% on images with lower quality, such as panoramic radiographs. This demonstrates that vertical misfit can be detected using routinely employed panoramic radiographs without the need for additional periapical x-rays. Additionally, given the retrospective nature of this study, existing panoramic radiographs were utilized for analysis. The use of panoramic radiography for evaluating prosthetic fit offers a more practical alternative to micro-CT in clinical environments, as it minimizes radiation exposure. This methodology is well-supported by similar studies in the literature, which have also favored panoramic-based validations for these clinical advantages [24,25].

It is noteworthy that among the architectures tested in our study, RegNetY-800MF (98.25%) was more successful than Transformer-based models such as Swin Transformer (78.07%) and DeiT (83.33%). This is consistent with the literature stating that while Transformer models generally perform highly on large datasets, CNN-based models (RegNet, ConvNeXt) yield more stable results in medical imaging tasks where data volume is limited. Unlike CNNs, which possess translation equivariance and extract local features effectively, Vision Transformers rely on global self-attention mechanisms. The inherent anatomical distortions, ghost images, and varying magnification rates in panoramic radiographs introduce structural noise that disrupts the global pixel relationships, making it difficult for ViTs to focus on micro-level gaps without massive training datasets [20,26,27]. Furthermore, the CBAM and ECA modules added to the model ensured that the model focused solely on the micro-gap in the implant neck region by suppressing the noisy background (bone trabeculation, superpositions) in the panoramic image. This was confirmed by the 100% Precision value obtained specifically for the Misfit class. In clinical practice, this indicates that there is no margin of error when the artificial intelligence identifies a case as a misfit, thereby preventing unnecessary prosthesis removal.

Similarly, Ayhan et al. reported that when CBAM was integrated into the YOLOv7 architecture in their study on the detection of caries under fixed prostheses on panoramic radiographs, the model’s performance increased [28]. The authors attributed this increase to the attention module improving feature maps and enabling more precise detection in noisy and metal-artifact areas. These findings align with our results, indicating that CBAM and other attention modules increase diagnostic accuracy by suppressing structural noise in panoramic images.

Fasih et al. reported the diagnostic accuracy of dentists as 93.3% in their study and found no statistically significant difference between AI and clinicians [19]. However, the 99.12% success rate in our study indicates that artificial intelligence surpasses the limits of the human eye (90–93% range in the literature). The high sensitivity of the proposed model in capturing deviations exceeding 10–150 μm, which are difficult for the human eye to detect on panoramic radiographs, suggests that this model could serve as a reliable clinical decision support system for non-expert dentists and epidemiological studies [6,7].

**Figure 5:**
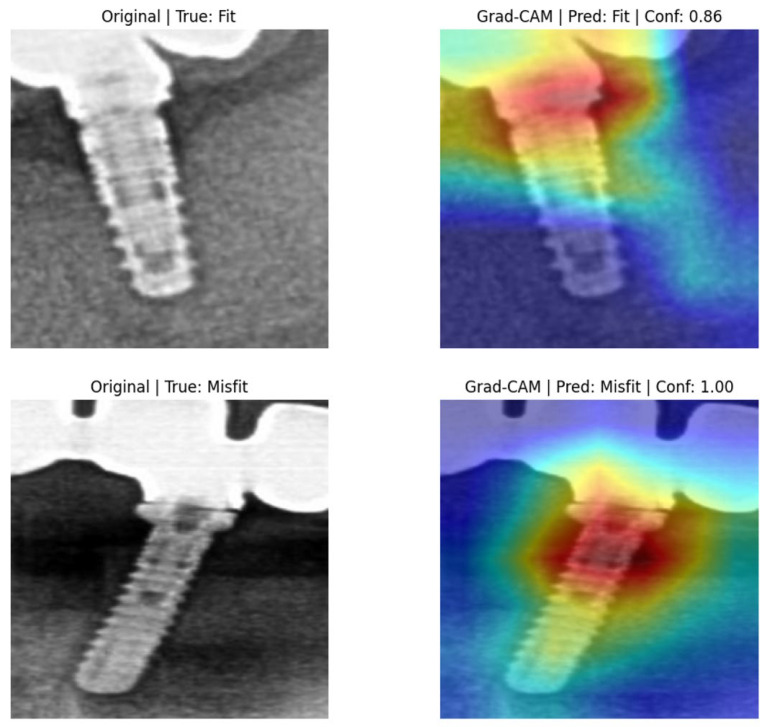
Grad-CAM examples for Fit and Misfit classes. The color range between blue and red represents the importance of the area the model is focusing on, from low (blue) to high (red).

To enhance the interpretability of the decision-making mechanism of the proposed model and to support the clinical reliability of the obtained performance, the Gradient-weighted Class Activation Mapping (Grad-CAM) method was used in this study. Since the black-box nature of deep learning models poses limitations in terms of clinical acceptance in medical image analysis, explaining which image regions the model focuses on when making decisions is of great importance. Therefore, the Grad-CAM method clarifies the model’s decision-making process by visualizing the spatial regions that contribute most to the classification process through heat maps [29]. The Grad-CAM visualizations obtained in this study were analyzed to reveal how the proposed hybrid architecture works in conjunction with attention mechanisms and which structural details it highlights. The resulting sample visualizations are presented in Figure 5.

Figure 5 shows that the proposed model, when determining fit and misfit images, primarily focuses on the abutment-prosthesis interface and the implant neck region. It is observed that high activation areas are concentrated in these regions, while the background bone tissue and adjacent anatomical structures contribute less. In fit samples, activations are more homogeneous and spread across the contact surface, while in misfit samples, localized and sharp concentrations are observed. The resulting different activation distributions demonstrate that the proposed model can distinguish not only global shape characteristics but also subtle structural discontinuities and density variations at the interface.

Recent advancements in AI-driven diagnostic tools have significantly accelerated the integration of automated workflows into routine clinical practice. Commercially available software platforms and state-of-the-art frameworks, such as 3Shape Automate and Dynasmile, have shown remarkable success in broad diagnostic and planning tasks, including automated cephalometric tracing, caries detection, and 3D implant positioning [30,31]. However, while these comprehensive systems excel in macro-level anatomical evaluations, the specific detection of micro-level discrepancies, such as vertical misfit at the implant-abutment interface on 2D panoramic radiographs, remains a specialized challenge that is often overlooked by general-purpose AI tools. The attention-enhanced hybrid RegNetY model proposed in this study addresses this specific gap. By isolating the critical micro-gaps and suppressing background noise, our targeted architecture offers a specialized solution that could potentially be integrated as an advanced sub-module within these broader commercial AI ecosystems.

Our study addresses some of the limitations of the study by Fasih et al. By including a different imaging technique, more deep learning models, and attention mechanisms, high success was achieved in detecting vertical misfit [19]. Nevertheless, this study has certain limitations. The study focused solely on vertical misfits, excluding horizontal misfits. Future studies are recommended to investigate horizontal misfits using 3D imaging techniques. The model should be tested on a larger dataset by incorporating data from different centers. The dataset was obtained from a single clinical center using a specific panoramic X-ray unit. Variations in image quality, contrast, and magnification ratios inherent to different panoramic devices (e.g., different manufacturers or sensor technologies) may impact the generalizability of the model. Furthermore, patient-related factors such as positioning errors, which are common in panoramic radiography, could introduce anatomical distortions that the model has not yet encountered. Future research involving multi-center datasets and diverse imaging equipment is necessary to validate the global clinical applicability of the proposed attention-enhanced framework. Furthermore, binary classification (fit vs. misfit) oversimplifies a continuous clinical parameter. Future studies should aim to quantify the exact amount of misfit in micrometers using regression-based deep learning models.

## 5. Conclusions

This study has demonstrated the high success of deep learning models for the detection of vertical misfit on panoramic radiographs. In particular, the hybrid RegNetY-800MF model enhanced with attention mechanisms (SE, CBAM, ECA) exhibited superior performance compared to both standard CNN and Transformer-based models, with an accuracy rate of 99.12%. In conclusion, it has been shown that even routine but lower-resolution imaging methods like panoramic radiographs can be transformed into a diagnostic tool when the appropriate architecture (RegNetY) and attention mechanisms are utilized.

## Figures and Tables

**Table 1:** Performance metrics comparison for all models.

Model	Accuracy	Macro-F1 Score	Macro Precision	Macro Recall
RegNetY-800MF	98.25	98.23	98.08	98.44
ConvNeXt-Tiny	96.49	96.46	96.30	96.88
EfficientNetV2-S	89.47	89.26	89.49	89.09
ResNet50	88.60	88.55	88.60	89.19
DeiT	83.33	83.30	86.23	85.16
Swin-Tiny	78.07	76.96	79.03	76.53
Proposed Model	99.12	99.21	100.00	98.44

**Table 2:** Class-based performance metrics comparison for proposed model.

Class	F1 Score	Precision	Recall
Fit	99.01	98.04	100.00
Misfit	99.21	100.00	98.44

**Table 3:** Performance metrics of the proposed model for fit–misfit classification with 95% confidence intervals.

Metrics	Value (95% CI)
Accuracy	99.12 (97.37–100.00)
Precision	98.45 (94.80–100.00)
Recall	100.00 (100.00–100.00)
F1-score	99.21 (97.33–100.00)

## Data Availability

Data used in this research are available upon reasonable request from the corresponding author.
